# Spinal Cord Syndrome Due to Extramedullary Epithelioid Hemangioendothelioma of the Thoracic Spine: A Case Report and Literature Review

**DOI:** 10.7759/cureus.58571

**Published:** 2024-04-18

**Authors:** Eliezer Villanueva-Castro, Sergio Ramírez-Aragón, Jorge Del Pino-Camposeco, Obet Canela-Calderon, Juan Antonio Ponce-Gómez, Juan Nicasio Arriada-Mendicoa

**Affiliations:** 1 Department of Neurosurgery, Instituto Nacional de Neurología y Neurocirugía Manuel Velasco Suárez, Mexico City, MEX; 2 Department of Neurosurgery, Hospital Central Militar, Mexico City, MEX

**Keywords:** radiation therapy, vascular bone tumor, spine surgery, spine tumor, epithelioid hemangioendothelioma

## Abstract

We report a 48-year-old male patient with spinal epithelioid hemangioendothelioma in T3 and T4 who began with symptoms of paresthesia in the lower limbs and distal weakness of the right lower limb, back pain, increased limitation in walking, urinary incontinence, and constipation. A safe maximum resection was performed, finding residual disease during the PET/CT scan, so it was decided to treat with radiotherapy, and there was a good response to this treatment. A literature review of epithelioid hemangioendothelioma of the thoracic spine was done which showed a mean age of presentation of 41 years and a male-female ratio of 1:0.53. The main symptom was pain, which was present in 100% of the patients, and wide surgery was performed in 56.8% of the patients, intralesional surgery in 31.8%, and biopsy in 11.4%. A total of 46.6% of patients received radiation therapy, and only 6.6% received chemotherapy. The patients had an average follow-up of 38 months. We recommend that extension studies such as PET/CT scans be performed after surgical resection. This can serve as a follow-up with hemangioendothelioma epithelioma patients about metastatic disease or residual disease that will guide us in giving adjuvant treatments, such as radiotherapy or chemotherapy, for better control of the disease.

## Introduction

Epithelioid hemangioendothelioma (EHE) is a rare vascular tumor, accounting for less than 1% of all vascular cancers [1]. It has an incidence rate of 0.038/100,000 per year and a prevalence of <1/1,000,000. It has a slight predominance in females [2], with a maximum incidence at 44 years of age [3].

The first description of EHE was in 1982 as a vascular neoplasm of bone and soft tissue, following a clinical course between a hemangioma and an angiosarcoma [4]. It is commonly seen in soft tissues but can also be in other organs, such as the lung, pleura, vessels, heart, liver, and bone [5]. Primary EHE of the bones and spines is rare and accounts for only 1% of all malignant bone tumors [6,7]. According to the World Health Organization’s classification of bone tumors, EHE is defined as a low-to-medium-grade malignant neoplasm that may involve the long bones of the limbs and axial skeleton, as well as the phalanges of the hands and feet [8].

The clinical presentation of the tumor lesion is variable; the diagnosis can be incidental. In symptomatic cases, the most common symptom is local pain (30%) or it can be presented as palpable mass (9%) and weight loss (9%) [5]. The most important symptom that exists at the time of diagnosis is back pain related to a vertebral fracture [7]. The imaging diagnosis can be seen in computed tomography (CT) as heterogeneous hypodensity with hyperdensity-pointed focuses and in magnetic resonance imaging (MRI) with heterogenic hypointensity in T1-weighted and hyperintensities in T2-weighted. In CT and MRI scans, the lesion has a heterogeneous highlight ranging from moderate to intense [9].

This report describes the case of a patient with thoracic spine EHE treated with spine surgery and adjuvant radiotherapy treatment who fully recovered from their symptoms. Additionally, a literature review was conducted of spine EHE and cases of primary EHE in the thoracic spine.

## Case presentation

A male patient, 48 years old, was referred to our department for surgical surgery. Past history included paresthesia in the lower limbs and distal weakness in the right lower extremity. Two months after this initial presentation, he had back pain, and progressive weakness in the left lower extremity was added, with a limitation to walking four months later. Six months after the onset of symptoms, loss of urinary sphincter control and constipation were added. Keeping all the above in mind, he was then referred for spinal surgery.

**Figure 1:**
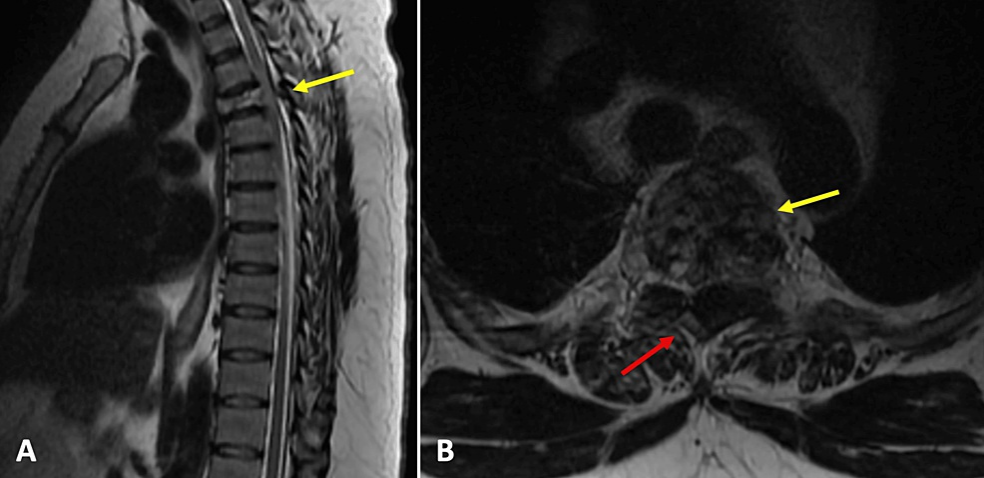
Pre-surgical magnetic resonance imaging (A) In the sagittal sections of T2-weighted MRI, a decrease in the signal of the T3 vertebral body lesion is observed, which is compressing the spinal cord (yellow arrow); (B) In the axial section of T2-weighted MRI, a lesion is observed that destroys the vertebral body (yellow arrow) and occupies the spinal canal, displacing neural tissue to the right (red arrow).

**Figure 2:**
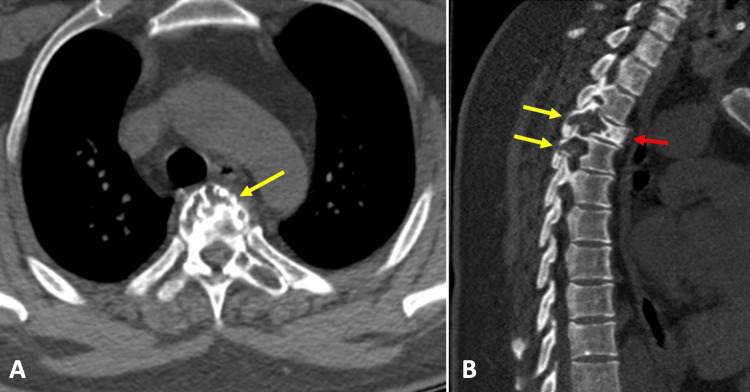
Pre-surgical computed tomography (A) Axial section shows a lytic lesion affecting the vertebral body of T3 (yellow arrow); (B) Sagittal section shows involvement of articular facets and laminae of T3 and T4 (yellow arrows) and decrease in T3 signal (red arrow).

The physical examination in our department revealed a strength of 1/5 in the pelvic extremities proximally and distally on the Daniels scale, patellar and Achilles hyperreflexia, and hypoesthesia with a sensory level at T6. There was a presence of urinary incontinence. In MRI studies, an extradural tumor lesion was observed that displaced the spinal cord and affected the vertebral body and articular facets at the level of T3 and part of the vertebral body of T4, as well as the presence of a tumor lesion in paravertebral muscles (Figure 1). In a CT scan, a lytic lesion was observed involving the body and articular facets of T3 and T4 (Figure 2).

**Figure 3:**
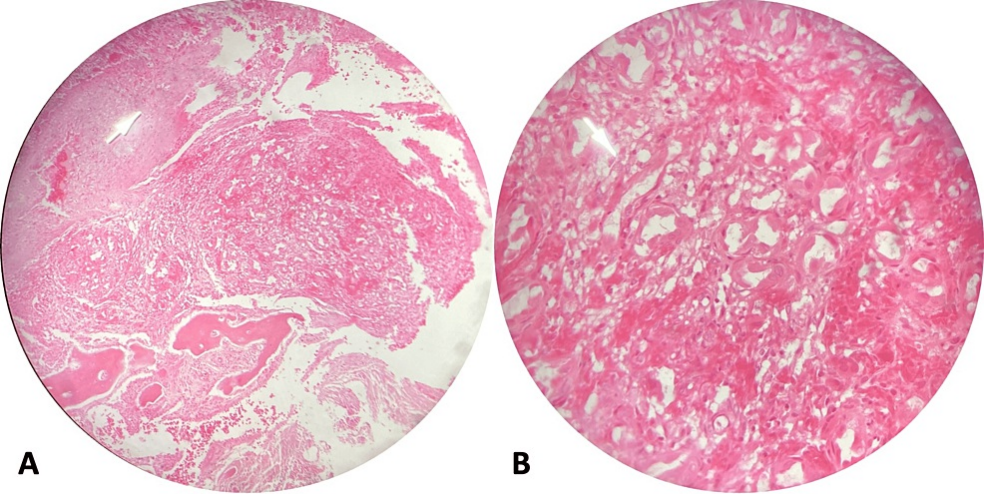
Histopathology study showing presence of areas of fibrosis with reparative bone resorption, characteristics of epithelioid hemangioendothelioma (white arrow).

Surgical treatment was performed with a posterior approach along the midline. A slight infiltration of muscle tissue was found due to the tumor that was completely resected, as well as a laminectomy with T3 corpectomy and resection of affected bone of T4. Fixation was performed with T1-T2 and T5-T6 bilateral transpedicular screws with two bars. The histopathology reported epithelioid hemangioendothelioma (Figure 3).

**Figure 4:**
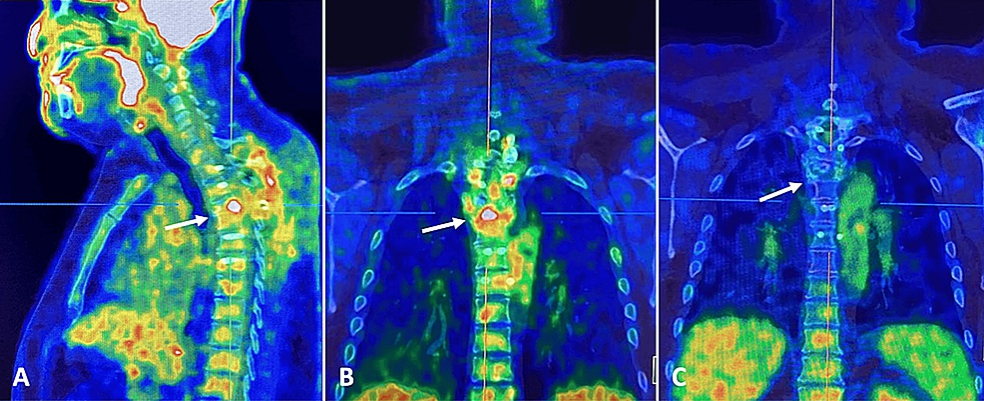
Post-surgical PET/CT study (A, B) PET/CT study before treatment with radiotherapy, in which hypermetabolism is observed with 18F-FDG uptake at the surgical site (white arrow). (C) Control PET/CT study one year after radiotherapy, where there is an absence of 18F-FDG hyper-uptake areas (white arrow). PET/CT: positron emission tomography/ computed tomography; 18F-FDG: 2-deoxy-2-(fluorine-18) fluoro-D-glucose.

During follow-up, positron emission tomography with 2-deoxy-2-(fluorine-18) fluoro-D-glucose integrated with computed tomography (18F-FDG PET/CT) was performed, with the presence of hypermetabolism at the surgical site. Metastatic lesions were ruled out (Figure 4). For this reason, he was approached by the radiotherapy service, and adjuvant therapy was administered with 37 Gy in 21 fractions. After six months of radiotherapy, the patient recovered bladder function, ambulation without alterations, muscle strength at 5/5 in the lower extremities without alterations in reflexes, and complete resolution of the spinal cord syndrome.

**Figure 5:**
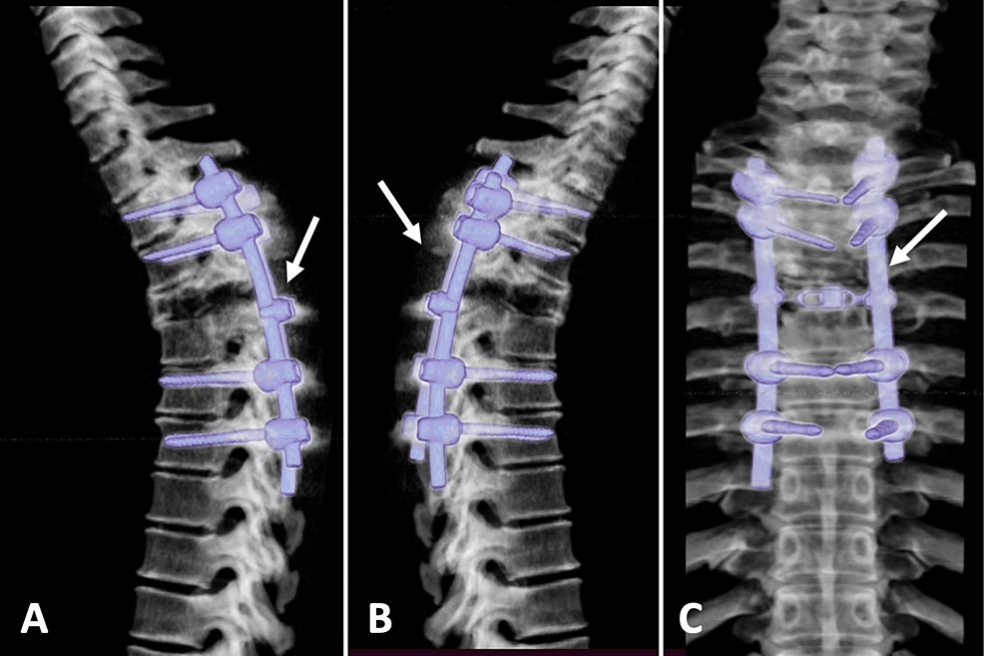
CT with post-surgical 3D reconstruction Lateral (A and B) and anteroposterior (C) views show adequate placement of the fixation system (white arrow).

The patient had control studies of MRI and CT of the dorsal column, where the presence of tumor recurrence was ruled out. With a well-placed fixation system (Figure 5), and PET/CT scans showing no recurrence, no areas of hypermetabolism were observed (Figure 4C). At 48 months of follow-up, the patient remained asymptomatic and had no evidence of disease.

## Discussion

Fractionated radiotherapy with 37 Gy in 21 fractions was chosen to supplement the treatment after a PET/CT scan for the current patient revealed residual disease at the surgery site, rather than searching for metastatic disease. After six months of treatment, the patient showed a considerable improvement in their clinical condition, and at 48 months of follow-up, there was no indication of the disease. Guy et al. used radiation therapy for an EHE of the spine using volumetric intensity-modulated arc radiotherapy on a tumor that could not be embolized or resected due to profuse bleeding [10]. The EHE received 54 Gy in 27 fractions, with a follow-up of 18 months. Their patient presented improvement in back pain without acute toxicity from radiotherapy.

A review of the literature on the primary EHE of the thoracic spine was done. The search was performed in PubMed with the terms “epithelioid hemangioendothelioma, spine.” Articles on the primary EHE of the thoracic spine were selected, and those that did not report patient information were excluded. Articles on multiple tumor locations in the spine and those that did not specify the primary tumor in the case of metastasis were excluded too. The search yielded a total of 27 authors and 45 patients. All cases of primary EHE of the thoracic spine were grouped based on the following data: author, year of publication, age, sex, location, symptoms, management, follow-up, and outcome. The data were analyzed, including this case, with these results: an average age of 41.15 years (range, 15-78 years) and a male-female ratio of 1:0.53 (30 males/16 females). In two patients, no information about symptoms was found but the remaining presented with pain. The mean follow-up was 38 months (Table 1).

**Table 1:** Primary epithelioid hemangioendothelioma of the thoracic spine or a greater thoracic location M: male; F: female; T: thoracic; WS: wide surgery; IS: intralesional surgery; RT: radiation therapy; CHT: chemotherapy; FU: follow-up; NED: no evidence of disease

Authors (year)	Case number	Age/Gender	Location	Symptoms	Treatment	Follow-up (months)	Outcome
Presenting case (2024)	1	48/M	T3-T4	Paresthesia, weakness in the lower limbs, back pain, limitation to walking, loss of urinary sphincter control, and constipation	IS + RT	46	Neurological improvement, NED
Ge et al. (2023) [11]	2	43/M	T8	Back pain, lower limbs hypoesthesia	WS	36	Without tumor recurrence
Chen et al. (2022) [12]	3	42/M	T2	Back pain, lower limbs hypoesthesia	WS	15	NED
	4	21/F	T9	Back pain, lower limbs hypoesthesia, and weakness	WS + RT	22	NED
	5	47/M	T6	Lower back pain, lower limbs hypoesthesia	WS	85	NED
	6	72/F	T3-T4	Lower back pain, lower limbs hypoesthesia, and weakness	Biopsy + RT	29	Partial regression
	7	15/M	T3	Back pain	WS	Loss to FU	Not specified
	8	73/M	T10	Back pain	WS	26	NED
	9	67/F	T5	Chest pain	WS	40	NED
Song et al. (2020) [13]	10	45/M	T2-T4	Not specified	Not specified	Not specified	Not specified
Zeng et al. (2020) [14]	11	42/M	T5	Thorax and low back pain	Biopsy	Not specified	Not specified
Slavnic et al. (2019) [15]	12	58/F	T4	Respiratory distress, difficulty walking and chest pain.	IS + RT after local recurrence	18	Local disease after first surgery. Then IS + RT. Currently stable disease.
Patel et al. (2018) [16]	13	49/M	T6	Back pain, lower limbs weakness, decreased sensory perception	WS + CHT+ RT	3	Right atrium metastasis that improved after chemotherapy and later with metastatic disease.
Guo et al. (2017) [17]	14	34/F	Not specified	Not specified	Excision	12	Alive with active disease
Albakr et al. (2017) [18]	15	34/M	T5	Back pain, ataxia, and left lower limb weakness	IS + RT	3	Pain disappearance and neurological improvement
Munier et al. (2017) [19]	16	65/M	T7-T9	Back pain	WS	58	Local recurrence after two years (IS + RT). Appearance of a new EHE in T11 (RT) ten months later. Stable disease two years later.
Das et al. (2017) [20]	17	24/F	T2-T4	Pain and neurological deficits	IS	6	Neurological improvement
Luzzati et al. (2015) [7]	18	28/F	T4-T5	Pain	WS	109	NED
	19	41/M	T1	Pain	WS	36	Dead (lung metastasis)
	20	66/F	T5-T6	Pain	RT + WS	124	NED
	21	17/F	T8	Pain	WS	90	NED
	22	41/M	T5	Pain	WS	30	NED
	23	53/M	T1-T3	Pain	WS	61	Local disease (37 months later)
	24	40/M	T7	Pain	WS	124	NED
Guy et al. (2014) [10]	25	48/F	T10	Back pain	Biopsy + RT	18	Stable
Kerry et al. (2012) [6]	26	25/M	T7	Back pain	IS + RT + CHT	2	Dead (lung, pleural, lymphonodular, and cutaneous metastasis)
Gomez-Arellano et al. (2012) [21]	27	19/M	T1,T3-T4	Back pain and paraparesis	Biopsy + Palliative RT	5	Lung and pleura metastasis
de Singly et al. (2011) [22]	28	78/M	T9	Periumbilical pain and weight loss	Surgery (vertebrectomy)	12	Colon metastasis
Ma et al. (2011) [23]	29	42/M	T3	Back pain	WS + RT	58	NED
	30	50/M	T8-T9	Back pain	WS + RT	48	NED
	31	22/F	T9	Back pain	IS + RT	34	Dead (metastasis)
Wang et al. (2009) [24]	32	22/F	T12	Low back pain, paresthesia	WS	Not specified	Not specified
Kopniczky et al. (2008) [25]	33	43/M	T10	Back pain, paraparesis, lower limbs hypoesthesia	WS	12	Neurological improvement, NED
Aquilina et al. (2005) [26]	34	17/M	T10	Nighttime back pain	WS	16	Local relapse
Themistocleous et al. (2005) [27]	35	23/F	T8	Back pain, paraplegia	IS + RT	26	NED
Abuzallouf et al (2005) [28]	36	41/M	T12	Low back pain radiating to the leg	IS + RT	48	NED
Aflatoon et al. (2004) [29]	37	74/F	T4-T5	Back pain, paresthesia	IS + RT	60	NED, back pain
	38	21/M	T12	Back pain	WS + RT	72	Dead (post-irradiation sarcoma)
	39	28/M	T1-T2	Back pain and paresis	IS + RT	4	Diffuse lung metastasis. Alive with active disease.
Evans et al. (2003) [30]	40	25/M	T11	Low back pain	IS + CHT	60	Dead (skull, femur, humerus, scapula, mediastinal lymph node, liver and lung metastasis
Faust et al. (2001) [31]	41	58/F	C7-T4	Pain, upper edema in the upper limbs, segmental sensory disturbance, Horner´s syndrome	IS	1	Dead (post-surgical complications)
Abrahams et al. (1992) [32]	42	34/M	T3	Back pain, hypoesthesia in the lower limbs	Biopsy	Not specified	Not specified
Tsuneyoshi et al. (1986) [33]	43	73/M	T10-L1	Back pain	IS + RT	Not specified	Not specified
	44	16/M	T9	Back pain	IS	48	Asymptomatic
	45	26/M	T11	Back pain	WS + RT	20	Metastasis
Maruyama et al. (1985) [34]	46	43/F	T3	Pain, neurological deficit	WS + RT	1	Neurological deficit resolved

Das et al. conducted a review of spinal EHEs in 2017 [20]. According to their review, spinal EHE occurs mainly in the thoracic spine (53%), 24% in the cervical spine, and 24% in the lumbar spine, affecting more frequently the population in the fifth decade of life, with greater involvement in men than in women. The most frequent clinical presentation was pain in 94% of patients, which is very similar to our review.

EHE is a rare tumor lesion that can affect tissues adjacent to the spinal cord as well as the vertebral column. Its growth can extend towards the spinal canal. Such involvement can simulate a clinical picture of spinal cord syndrome. However, it is important to consider differential diagnoses of conditions that affect the spine including myeloma, sarcomas, metastatic lesions, schwannoma, meningioma, and other vascular tumors [1].

According to a study by Dang et al., the main symptom of primary spinal tumors was local pain in 77% of their cases. Almost 50% of their patients did not experience other symptoms. The next most common symptom was neurological deficit in 45% of cases, depending on the extent and level of the tumor [35]. Weakness and sensitive disturbances in the upper limbs are found in cervical tumors, and patients with thoracic and lumbar tumors report spasticity, weakness, and sensory disturbances in the lower limbs [36]. Also, cauda equina syndrome is caused when compression at the levels of L3/L4 and L4/L5 occurs [37].

For the imaging diagnosis of EHE, MRI and CT studies are ideal. MRI helps us to better see the infiltration in the soft tissues and identify the vascular tissue. CT is better to observe changes in the bone of the spine. EHE presents as an expansive osteolysis with poorly defined limits and surrounding soft tumor tissue. Pathological compression fractures may be present. MRI shows characteristics of a vascular tumor with isointensity on T1-weighted, hyperintensity on T2-weighted, and contrast enhancement [12]. 

In the diagnostic approach, some authors recommend biopsies of soft tissue tumors for histological and immunohistochemical recognition of the lesion, planning surgical resection with the histological result, and, if necessary, neoadjuvant therapy [38]. In our patient, it was not an option due to the spinal condition and the acute spinal syndrome with the presence of sphincter alterations. A safe maximum resection with complete spinal decompression was initially planned to give him the greatest recovery benefit and reduce the risk of neurological conditions due to chronic evolution. 

There is no specific treatment guideline for the treatment of spinal EHE, but the ideal treatment is a complete resection of the tumor. Preoperative embolization, radiation therapy, and chemotherapy can be performed [11]. In our review, we found that wide surgery was performed on 56.8% of the patients, intralesional surgery on 31.8%, and biopsy on 11.4% (Table 1). A total of 46.6% of patients received radiation therapy, and only 6.6% received chemotherapy with bevacizumab and doxorubicin. Wide surgery was initially recommended, but in very complex cases or eventualities during surgery, it was decided to perform intralesional surgery and, in some cases, adjuvant treatment.

In some cases, preoperative embolization may not be successful, or surgery may be necessary due to abundant bleeding or the complexity of tumor resection. In these cases, it is necessary to perform radiation therapy as a treatment [10,12]. Gómez-Arellano et al. reported a patient with three-level thoracic EHE with metastasis to the pleura and lung who received only palliative radiation therapy due to the rapid progression of the disease [21].

Guy et al. showed that treatment with volumetric intensity-modulated arc radiotherapy for EHE can be curative and help preserve critical organs [10]. Treatment with bevacizumab is a useful chemotherapy treatment for metastatic or recurrent disease [16,20]. The EHE has moderate activity for 18F-FDG PET/CT, and due to its intrinsic metabolic activity, it represents a good option as an extension study in search of residual or metastatic disease [13,39].

## Conclusions

The literature review showed that patients with primary EHE of the thoracic spine have an average age of 41 years, a higher presentation in men, and pain is the main symptom. Wide surgery is performed in most cases, followed by intralesional surgery, and the most common concomitant treatment is radiation therapy. There is no specific clinical or surgical guideline for the treatment of this tumor.

With the data from the follow-up, we can presume that it is very important to carry out a rapid treatment of the spinal compression due to the irreversible complications. As we demonstrated in this case, having a diagnosis of EHE after a safe maximum resection is not enough, and other studies must be carried out, including PET/CT scans, to rule out residual tumor or metastasis. Prompt diagnosis may lead physicians to provide the most appropriate treatment for the patient, with radiotherapy or adjuvant chemotherapy, with a complete follow-up that includes imaging studies due to the high risk of recurrence.
